# Out-of-equilibrium phonons in gated superconducting switches

**DOI:** 10.1038/s41928-022-00721-1

**Published:** 2022-02-28

**Authors:** M. F. Ritter, N. Crescini, D. Z. Haxell, M. Hinderling, H. Riel, C. Bruder, A. Fuhrer, F. Nichele

**Affiliations:** 1 IBM Quantum, IBM Research—Zurich, Rüschlikon, Switzerland; 2 Department of Physics, University of Basel, Basel, Switzerland

**Keywords:** Superconducting properties and materials, Superconducting devices

## Abstract

Recent experiments have suggested that superconductivity in metallic nanowires can be suppressed by the application of modest gate voltages. The source of this gate action has been debated and either attributed to an electric-field effect or to small leakage currents. Here we show that the suppression of superconductivity in titanium nitride nanowires on silicon substrates does not depend on the presence or absence of an electric field at the nanowire, but requires a current of high-energy electrons. The suppression is most efficient when electrons are injected into the nanowire, but similar results are obtained when electrons are passed between two remote electrodes. This is explained by the decay of high-energy electrons into phonons, which propagate through the substrate and affect superconductivity in the nanowire by generating quasiparticles. By studying the switching probability distribution of the nanowire, we also show that high-energy electron emission leads to a much broader phonon energy distribution compared with the case where superconductivity is suppressed by Joule heating near the nanowire.

## Abstract (web summary)

Suppression of superconductivity in metallic nanowires due to a gate voltage can be linked to the relaxation of high-energy electrons and not to the presence of electric fields at the superconductor surface.

## Main

It is generally thought that metallic nanostructures are not affected by electric fields, as long as their size is larger than the corresponding screening length, which is typically below 1 nm. Recent experiments^1–6^ have, however, shown that gate voltages can have a dramatic impact on the superconducting properties of metallic devices, including the ambipolar quenching of the critical current. The microscopic mechanism responsible for this behaviour has sparked debate. First, it was suggested that an electric field can penetrate a superconducting film up to the London penetration depth^1^. Second, it was proposed that an electric field might perturb the polarization of atomic orbitals at the metal surface, and this would affect the superconducting properties in the bulk^7,8^. Third, studies of the switching probability distribution (SPD) in metallic nanowires suggested an interplay between an electric field and superconducting phase slips^5^.

We have previously reproduced the most distinctive features of these experiments using titanium nitride (TiN), niobium and titanium nanowires^9^. In our samples, the critical current suppression was always accompanied by a current flowing between the gate and nanowire. In these experiments, the gate current is carried by electrons with energies of several electronvolts, which is orders of magnitude larger than the superconducting energy gap in the nanowires. We concluded that the emission of relatively few electrons leads to an avalanche of quasiparticles, which effectively quench the critical current^10^. This hypothesis was supported by tunnelling spectroscopy experiments^11^, which highlighted a non-thermal increase in quasiparticle population as a gate voltage was applied. Further work also demonstrated a correlation between the onset of gate currents and suppression of superconducting properties^12,13^. However, open questions remain. For example, in a scenario where the injection of high-energy electrons controls the critical current suppression, a marked asymmetry would naively be expected between injecting high-energy electrons into the nanowire (negative gate voltage) and extracting electrons from the nanowire at the Fermi energy (positive gate voltage), as well as having them relax either in the substrate or in the gate electrode. Unravelling the microscopic mechanisms behind these observations could prove valuable in the development of technological applications of the phenomenon, such as the realization of voltage-controlled superconducting switches and resonators.

In this Article, we show that the quenching of superconductivity in metallic nanowires can be linked to the relaxation of high-energy electrons, and not to the presence of electric fields at the superconductor surface. In particular, we examine the effect of high-energy electrons flowing into the nanowire, out of the nanowire and between two remote gate electrodes in the vicinity of the nanowire. Detailed measurements reveal that superconductivity is most efficiently suppressed when a current is injected into the nanowire. However, a qualitatively similar critical current suppression is observed when high-energy electrons flow near the nanowire, without any current or electric field directly reaching the nanowire itself. The non-local nature of the observed effect is consistent with the energy relaxation of electrons by phonon emission in the substrate. Due to their relatively high energy, phonons generate quasiparticles in the superconductors and efficiently quench the critical current in our devices. At cryogenic temperatures, phonons can propagate over considerable distances in the crystalline silicon substrate before thermalizing. The effect is, thus, distinct from the situation where a local temperature increase is produced by a resistive heater. Our observations question the existing interpretations and theories based on electric fields, and provide an insight into the complex interactions between out-of-equilibrium phenomena in solids and the performance of superconducting hardware.

## Critical current suppression and electric fields

Seven TiN nanowires on Si substrates were investigated during this work. All the nanowires had a length of 2 μm, width of 80 nm and height of 20 nm. At low temperatures, the devices showed critical currents *I*_C_ between 42 and 45 μA, retrapping current *I*_R_ = 1.0 μA and normal-state resistance *R*_N_ ≈ 1,750 Ω, consistent with a previous work^9^. The uniformity of these values demonstrates that the nanowires were homogeneous and not characterized by accidental weak links. The large difference between *I*_C_ and *I*_R_ indicates substantial self-heating in the normal state, together with limited heat extraction via the leads or the substrate, typical of metallic nanowires^14,15^. Further details on sample fabrication and basic characterization are reported in another study^9^ and in Methods. Here we present the results from four devices, referred to as devices A1, A2, B and C. Extended data and three additional devices, used as references, are shown in more detail in Supplementary Figs. 1–3.

**Fig. 1:**
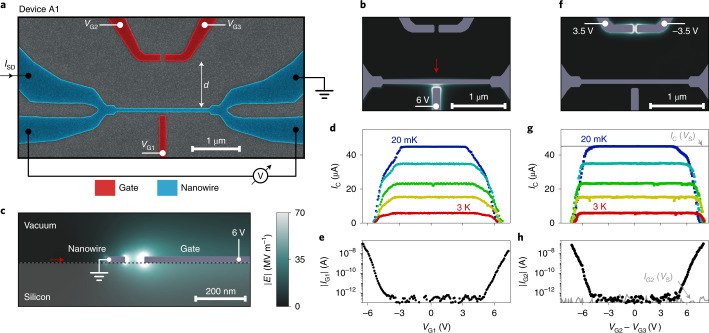
Basic device characterization and electric-field simulation. **a**, False-colour scanning electron micrograph of device A1, with a simplified measurement configuration. The nanowire under investigation is depicted in blue and the gates in red. **b**, Finite element simulation of the electric-field magnitude ∣*E*∣ for *V*_G1_ = 6 V. We show a slice of the 3D simulation on a plane elevated 10 nm from the Si substrate. **c**, Same as in **b**, but for a plane perpendicular to the substrate and intersecting gate 1. The red arrow indicates the direction of the cut in **b**. **d**, Critical current *I*_C_ in device A1 as a function of gate voltage *V*_G1_ for temperatures *T* of 20 mK (blue), 1.5 K, 2.1 K, 2.5 K and 3.0 K (red). **e**, Gate current *I*_G1_ as a function of *V*_G1_ measured at *T* = 20 mK simultaneously to the data in **d**. **f**, Finite element simulation as in **b**, but calculated for gate voltage difference *V*_G2_ − *V*_G3_ = 7 V. **g**, Critical current *I*_C_ in device A1 as a function of *V*_G2_ − *V*_G3_ for temperatures as in **d** (markers), together with *I*_C_ as a function of *V*_S_ = 2*V*_G2_ = 2*V*_G3_, representing twice the voltage simultaneously applied to both gates (grey line). **h**, Current *I*_G2_ flowing from gate 2 as a function of voltage difference *V*_G2_ − *V*_G3_. In this configuration, *I*_G2_ = −*I*_G3_ within the experimental error. Gate current *I*_G2_ as a function of *V*_S_ is shown in grey.

Figure 1a shows a false-coloured scanning electron micrograph of device A1, together with the schematic of the measurement configuration. Device A1 consists of a nanowire (blue) and three gates (red). Gate 1, controlled by voltage *V*_G1_, was separated from the nanowire by a gap of 80 nm. Gates 2 and 3, controlled by voltages *V*_G2_ and *V*_G3_, respectively, were separated from each other by 80 nm and from the nanowire by a distance *d* = 1 μm. A similar device, named device A2, had *d* = 80 nm (Supplementary Fig. 1).

We first discuss the response of device A1 to a side-gate voltage *V*_G1_, similar to previous work^1,9,11^. The electric-field distribution in this configuration was calculated using three-dimensional (3D) finite element simulations (Methods). Figure 1b shows the field magnitude ∣*E*∣ on a plane 10 nm above the substrate for *V*_G1_ = 6 V. Figure 1c represents ∣*E*∣ on a plane perpendicular to both substrate and wire axis, and intersecting the gate (Fig. 1b, red arrow). To better highlight the field distribution, the colour scale was saturated to ∣*E*∣ = 70 MV m^−1^. The highest ∣*E*∣ in our simulations was below ∣*E*∣ = 500 MV m^−1^, which is several orders of magnitude smaller than typical electric fields required to perturb superconductivity in a metallic device^16–18^. Figure 1d shows the experimentally measured *I*_C_ as a function of *V*_G1_, for temperatures ranging from 20 mK (blue) to 3 K (red). Figure 1e shows the gate current *I*_G1_ simultaneously measured to the data in Fig. 1d. Consistent with previous observations^9,12^, the decrease in *I*_C_ was correlated to the onset of *I*_G1_, and the initial decrease in *I*_C_ took place for *I*_G1_ < 1 pA. Furthermore, ∣*I*_G1_∣ was found to exponentially increase with *V*_G1_ and to be approximately symmetric around *V*_G1_ = 0.

We now discuss the dependence of *I*_C_ on a differentially applied voltage *V*_G2_ − *V*_G3_, with *V*_G2_ = −*V*_G3_. Figure 1f shows the numerically computed electric field for *V*_G2_ − *V*_G3_ = 7 V. As expected, ∣*E*∣ is strongly confined between gates 2 and 3. If superconductivity in the nanowire were controlled by the electric fields, this configuration should result in negligible effects on *I*_C_. Strikingly, quenching of the supercurrent occurred even in this situation (Fig. 1g). Figure 1h shows the current *I*_G2_ flowing from gate 2 (we found that *I*_G2_ = −*I*_G3_ within the experimental error). Remarkably, the suppression of *I*_C_ was strongly correlated to the onset of *I*_G2_, despite no measurable gate current reaching the nanowire and negligible electric fields between the gate and nanowire.

To test whether residual electric fields were relevant, we also measured *I*_C_ with gates 2 and 3 biased at the same voltage (*V*_G2_ = *V*_G3_). In Fig. 1g, we plot *I*_C_ as a function of the quantity *V*_S_ = 2*V*_G2_ = 2*V*_G3_ (Fig. 1g, solid grey line) as, at any one point in this plot, the absolute voltages ∣*V*_G2_∣ and ∣*V*_G3_∣ on the gate electrodes are identical and the absolute value of the electric field ∣*E*∣ reaching the nanowire is similar. More specifically, we estimate ∣*E*(*V*_G2_ = *V*_G3_)∣ ≳ ∣*E*(*V*_G2_ = −*V*_G3_)∣ at the nanowire surface. Nevertheless, no current was detected between the gates and nanowire for symmetrically applied gate voltages (Fig. 1h, grey curve) and *I*_C_ was not perturbed. These results further corroborate our findings that high-energy electrons, and not electric fields, are responsible for the suppression of *I*_C_. Similar results obtained with device A2 are presented in Supplementary Fig. 1.

Overall, experiments and numerical simulation presented in Fig. 1 demonstrate that the suppression of superconductivity takes place irrespective of the electric fields at the nanowire surface. Instead it requires the flow of high-energy electrons in the surroundings of the device. This is the first conclusion of our work.

## Role of substrate

**Fig. 2:**
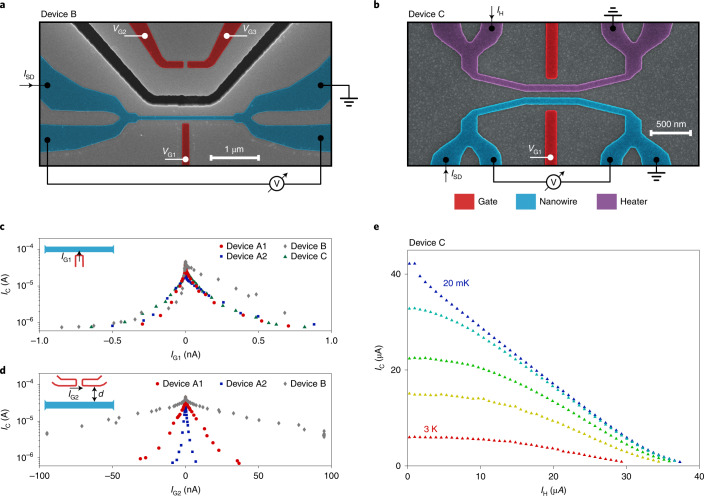
Additional devices. **a**, False-colour scanning electron micrograph of device B. The nanowire is depicted in blue; the gates, red; and the trench, black. The trench is 510 nm deep, 200 nm wide and has a total length of 80 μm. **b**, False-colour scanning electron micrograph of device C. The nanowire under investigation is depicted in blue; the gates, red; and the heater nanowire, purple. **c**, Plot of critical current *I*_C_ as a function of gate current *I*_G1_ for all the devices presented in the main text. **d**, Plot of *I*_C_ as a function of the remote gate current *I*_G2_ for devices A1 (*d* = 1 μm), A2 (*d* = 80 nm) and B (*d* = 1 μm plus a trench). **e**, Critical current in device C as a function of heater current *I*_H_ for temperatures as in Fig. 1d.

The remote action of *V*_G2_ − *V*_G3_ on *I*_C_ points to the existence of an efficient energy transfer mechanism triggered by the flow of *I*_G2_. We now analyse the origin of this remote action more carefully using devices B and C (Fig. 2a,b, respectively). Device B is identical to device A1, except for the presence of a 510-nm-deep, 200-nm-wide, 80-μm-long trench etched into the substrate between the remote gates and nanowire. Device C consists of two parallel TiN nanowires separated by a distance of 80 nm. Each nanowire was controlled by a nearby gate (Fig. 2b, red). We measured the critical current of one of the two nanowires (Fig. 2b, blue), whereas the second one (Fig. 2b, purple) was set in the resistive state and was traversed by a d.c. current *I*_H_, resulting in Joule heating, similar to another work^19^.

Figure 2c,d summarizes the behaviour of our devices in terms of *I*_C_ as a function of *I*_G1_ and *I*_G2_, respectively. The full dataset is presented in Supplementary Figs. 1 and 2. The dependence on *I*_G1_ (Fig. 2c) is similar in all the devices, with a faster suppression of *I*_C_ for *I*_G1_ < 0. Due to the exponential dependence of *I*_G1_ on *V*_G1_, this asymmetry is hard to spot in Figs. 1d,e. We further notice that device B (grey diamonds) exhibited a particularly slow decay of *I*_C_ for *I*_G1_ > 0. We will discuss the possible causes for this asymmetry below. Figure 2d reveals that *I*_G2_ is considerably less effective in suppressing *I*_C_ than *I*_G1_; furthermore, device A2 (blue squares; *d* = 80 nm) was six times more efficient than device A1 (red circles; *d* = 1 μm), which was six times more efficient than device B (grey diamonds; *d* = 1 μm plus an etched trench). In the case of device B, the maximum *I*_G2_ allowed in our setup (100 nA) was not sufficient to reach *I*_C_ = 0. Altogether, these results demonstrate that most of the remote action of *I*_G2_ on *I*_C_ is mediated by the substrate, that is, the high-energy electrons relax by emitting phonons, which travel through the substrate and affect superconductivity in the nanowire. This is the second main conclusion of our work.

## Comparison to Joule heating

We now discuss the properties of the generated phonons in more detail. In particular, we compare their effect on *I*_C_ with that of the heat generated by a resistive conductor placed 80 nm from the superconducting nanowire. These experiments were performed with device C (Fig. 2b). The dependence of *I*_C_ on heater current *I*_H_ is shown in Fig. 2e for various temperatures. As expected, Joule heating eventually resulted in the suppression of *I*_C_. However, the current required to reach *I*_C_ = 0 was several orders of magnitude higher than in the configurations where a gate voltage was applied.

**Fig. 3:**
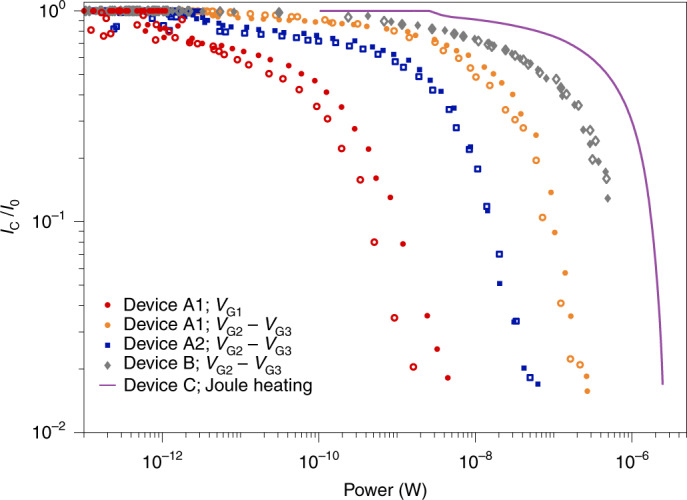
Comparison of switching power. Normalized critical current *I*_C_/*I*_0_ as a function of input power for various devices and experimental configurations. Full and empty markers define the positive and negative gate polarity, respectively. The solid purple line indicates the dependence as a function of Joule heating in device C.

Figure 3 provides a comparison between the devices presented above in terms of the suppression of normalized critical currents *I*_C_ as a function of dissipated power. For each measurement configuration, we distinguished the case of a positive and negative voltage bias with full and empty markers, respectively. Curves at higher temperatures are shown in Supplementary Fig. 4. The critical current is more efficiently suppressed when voltage bias *V*_G1_ is applied to a gate directly facing the nanowire (red dots). In this case, the dissipated power is calculated as *I*_G1_*V*_G1_. When a remote current *I*_G2_ flows, the power is calculated as *I*_G2_(*V*_G2_ − *V*_G3_). Suppressing *I*_C_ by means of Joule heating with a resistive conductor (Fig. 3, purple line) required a considerably higher power ${I}_{{{{\rm{H}}}}}^{2}{R}_{{{{\rm{N}}}}}$ than the other configurations. As noted above, the dependence on *I*_G1_ (Fig. 3, red circles) shows a difference between the positive and negative gate polarity, with the negative polarity being 2.5 times more power efficient in suppressing *I*_C_ compared with the positive one.

**Fig. 4:**
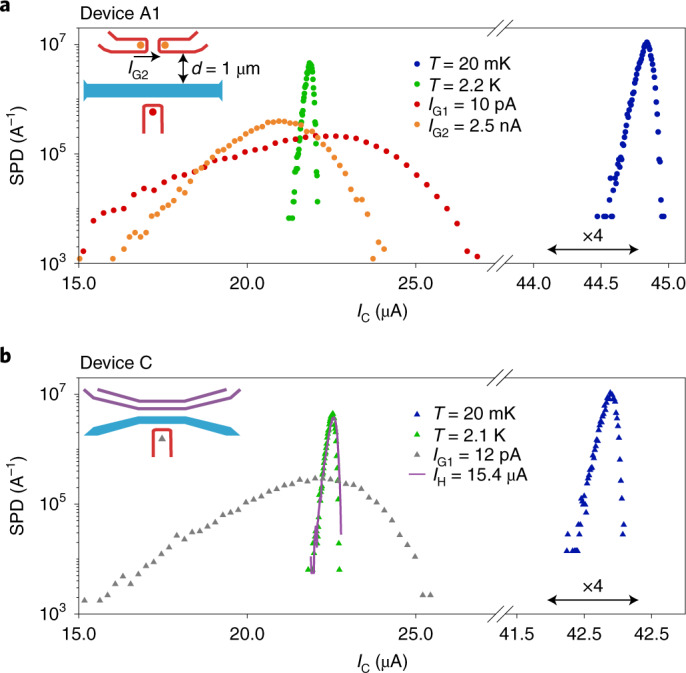
Comparison of SPDs. **a**, SPD in device A1 as a function of source–drain current *I*_SD_. The blue and green circles are obtained at zero gate voltage and for *T* = 20 mK and *T* = 2.2 K, respectively. The red circles are obtained for gate current *I*_G1_ = 10 pA (*V*_G1_ = 5.85 V) and the orange dots, for remote gate current *I*_G2_ = 2.5 nA (*V*_G2_ − *V*_G3_ = 7.25 V). Note that the horizontal axis is interrupted and the high-current region is horizontally expanded by a factor of four. **b**, Same data as in **a**, but for device C. The blue and green triangles are obtained at zero gate voltage and for *T* = 20 mK and *T* = 2.1 K, respectively. The grey triangles are obtained for *I*_G1_ = 12 pA (*V*_G1_ = 5.2 V) and the purple line is obtained for a heater current *I*_H_ = 15.4 μA.

We have shown that Joule heating is orders of magnitude less efficient in suppressing *I*_C_ of our nanowires than a current of high-energy electrons. In addition to these quantitative differences, we gain a further insight from the SPDs of our devices. The SPD is the probability of switching from the superconducting to resistive state to occur per unit of source–drain current. The SPD has proven to be a powerful tool to study Josephson junction and metallic nanowire properties that are hard to access with standard transport measurements^5,20^. Figure 4a,b shows the SPDs of devices A1 and C, respectively, under various experimental conditions. For these experiments, the source–drain current was swept 20,000 times from 0 to 49 μA. For each sweep, the source–drain current value at which a switch to the resistive state occurred was recorded. At low temperature and zero gate voltage, device A1 exhibited a sharp SPD (Fig. 4a, blue markers), with a standard deviation *σ*_*I*_ = 47 nA. At a temperature of 2.2 K (Fig. 4a, green markers), the SPDs had their maximum at half of the low-temperature *I*_C_ value, with *σ*_*I*_ = 100 nA. A more detailed analysis (Supplementary Fig. 5) revealed that the switching mechanisms at 20 mK and 2.2 K are consistent with quantum phase slips and thermal fluctuations, respectively. Much broader SPDs were obtained by applying a gate leakage current *I*_G1_ = 10 pA (Fig. 4a, red markers), with *σ*_*I*_ = 2.0 μA. The finding that the application of a gate voltage results in much broader SPDs than increasing the bath temperature (for equal suppression of *I*_C_) is consistent with the observations elsewhere^5^. However, we show that a similarly broad SPD is also obtained by applying a remote current *I*_G2_ = 2.5 nA (Fig. 4a, orange circles; *σ*_*I*_ = 1.2 μA), which is without any electric field or current reaching the nanowire. Using device C (Fig. 4b), we compare the SPD obtained when *I*_C_ is suppressed by 50% either by Joule heating (solid purple line) or by increasing the bath temperature to 2.1 K (green triangles). The two results are indistinguishable, indicating that a resistive heater indeed affects the superconductivity in the same way as an increase in the bath temperature, but in a totally different manner than a current of high-energy electrons (grey triangles). Because of the difference between the SPDs obtained at a high temperature (green markers) and finite gate voltage (red markers), another study^5^ excluded the presence of electrical currents. This conclusion was, however, reached under the assumption that a gate current causes heating similar to an increase in the bath temperature. Our results demonstrate, instead, that a current of high-energy electrons perturbs the superconducting properties of nanowires in a way that is qualitatively and quantitatively distinct from a bare temperature increase, even if the current does not flow into the nanowire but only in its surroundings. This is the third main conclusion of our work.

## Nature of generated phonons

Our observations are consistent with the phenomenology of phonon generation by hot electrons in the substrate. First, we note that phonons with energies above the superconducting gap (500 μeV for TiN (ref. ^21^)) are well known to affect superconducting devices^22–25^. Second, electrons accelerated by high electric fields in Si undergo a series of relaxation events over timescales below 1 ns and on mean free paths below 10 nm. Such relaxation most probably happens by the emission of optical and acoustic phonons^26–29^. Phonons in Si have a maximum energy of the order of 50 meV, which means that a single electron with an energy of a few electronvolts can generate a large amount of phonons^30,31^ as it travels between two metallic electrodes. At temperatures below 3 K, phonons in Si have long mean free paths (up to 1 μm (refs. ^32,33^)) and even longer thermalization lengths. It is, therefore, expected that the emitted phonons reach the nanowire in an out-of-equilibrium state^34,35^.

The electronic mean free path in Si decreases as ∣*E*∣ increases^26,27^, resulting in intense phonon emission close to the metal electrodes, independent of the gate voltage polarity (Fig. 1c)^30^. This may be the reason for the more efficient suppression of *I*_C_ when a current is either injected or extracted from the nanowire (Fig. 2c) compared with the case where a current flows between two gates near the nanowire (Fig. 2d, device A2). Furthermore, the suppression of *I*_C_ by a fixed factor requires 2.5 times less power for *V*_G1_ < 0 (Fig. 3) compared with *V*_G1_ > 0. This could indicate that electrons reaching the nanowire are not completely thermalized, and can still generate a sizeable number of quasiparticles via electron–electron interactions in the nanowire^10,36,37^. Assuming the phononic contribution is similar for both gate polarities (that is, phonon emission is isotropic), we estimate that more than half the suppression of *I*_C_ for *V*_G1_ < 0 is due to electron–electron interaction. Future work might use more complex geometries to map out angular anisotropies in the phonon emission and absorption processes.

The broadening of SPDs with gate voltage is consistent with the nanowire being subject to extremely energetic events, capable of suppressing superconductivity even at small source–drain currents. The characteristic energy spread of such events can be quantified by the Kurkijavi power law^20^, which allows one to relate the width of the SPD to the effective energy *E*_eff_ (Supplementary Section 5). As shown by the red dots in Fig. 4a, we obtain *E*_eff_ ≈ 8.6 meV, consistent with the idea that the energy of a leakage electron (7 eV) dissipates in successive scattering events in the substrate before reaching the nanowire. In the case of a remote current (Fig. 4a, orange dots), we get *E*_eff_ ≈ 6.3 meV, indicating that, on average, the phonons thermalize more over the longer distance. A possible framework for analysing such SPDs in Josephson junctions subject to high-energy electrons was recently proposed elsewhere^38^.

In device B, we noticed an anomalously large asymmetry in the parametric plot of *I*_C_ versus *I*_G1_ (Fig. 2c). With three reference devices (Supplementary Fig. 3a), we confirmed that such an asymmetry is a robust feature that arises following the fabrication steps required to etch trenches into the substrate (Methods). Similarly, the efficiency of the remote action of *I*_G2_ slightly decreased after additional fabrication, even when trenches were not etched (Supplementary Fig. 3b). Interestingly, no other sample parameters were affected by the additional fabrication steps. These results suggest that some of the out-of-equilibrium processes taking place in our device are sensitive to the surface treatment of the samples. Measuring device B, we have shown that out-of-equilibrium phonons are primarily responsible for the remote action of *I*_G2_ on *I*_C_. However, our work does not exclude the presence of additional energy relaxation mechanisms that contribute, together with phonons, to the suppression of *I*_C_ (such as photon emission). Previous works detected photons in a variety of devices as a result of tunnelling events^39–43^ as well as bremsstrahlung and carrier recombination of high-energy electrons^44,45^. It is also well known that superconducting nanowires^46^ and Josephson junctions^47^ are highly sensitive to the impact of high-energy photons. Both phonon and photon transport may be affected by the additional fabrication steps for trenching, for example, by a change in surface roughness or dielectric properties. The relative contribution of phonons and photons is estimated by comparing the response of devices A1 and B to *V*_G2_ − *V*_G3_ (Fig. 3). Device B required a six times higher power to reach the same *I*_C_/*I*_0_, indicating that the trench blocks five-sixth of the power that would have been otherwise absorbed by the nanowire. If we assume that any photonic contributions are unaffected by the trench, we can calculate an upper bound on such a contribution in that it must be smaller than one-fifth of the phononic contribution. Note that the reduced power reaching the nanowire in device B could also be carried by phonons, which—if travelling deep in the substrate—are also not affected by the trench.

## Conclusions

We have reported a comprehensive study of the mechanism responsible for the suppression of critical currents in metallic nanowires in the presence of large gate voltages. We have shown that previously reported features, which were attributed to the electric field on the superconductor, can be obtained in the absence of electric fields. Our data indicate that critical currents are suppressed as a consequence of the relaxation of high-energy electrons, either in the substrate or in the electrodes. Our results also elucidate the mechanism behind the ambipolar suppression of *I*_C_ as a function of gate voltage (Fig. 1d), which was not fully explained in previous works^9,11,12^. The ambipolar suppression of *I*_C_ requires an approximately symmetric gate current (which is experimentally observed (Fig. 1e)) as well as an efficient energy equilibration mechanism between the gate and nanowire. Energy equilibration is dominated in our devices by energetic phonons spreading through the substrate over distances in excess of 1 μm. Although this remote action may pose a limit to the device integration density, it could also open new paths for device design. For example, it could be used to develop efficient superconducting switches^48–51^ that do not require the injection of electrons into the switching element, but are instead mediated by high-energy phonons that are guided towards a switching element. It also opens new possibilities to investigate the interplay between out-of-equilibrium phenomena, resulting quasiparticle generation and superconducting quantum hardware.

## Methods

### Sample fabrication

A 20-nm-thick TiN film was sputtered on a Si substrate. The Si substrate used for this work was intrinsic and became insulating at temperatures below 100 K. Before TiN deposition, the Si chip was immersed in a buffered hydrofluoric acid (HF) solution for the removal of native oxides. The TiN film showed a critical temperature of 3.7 K and a resistivity of 68 Ω sq^–1^. Devices were defined by electron-beam lithography on a negative hydrogen silsesquioxane resist and dry etching in HBr plasma. The resist was then removed by immersion in HF. The devices were contacted by Ti/Au bond pads defined by optical lithography and metal evaporation. Some devices were further processed after the deposition of bond pads. In this case, 2 nm Si_3_N_4_ and 210 nm SiO_2_ hard mask were deposited by atomic layer deposition and plasma-enhanced chemical vapour deposition, respectively. A trench was defined in the hard mask with electron-beam lithography and CSAR AR-P 6200.09 resist, standard development and reactive ion etching of the SiO_2_ layer. The Si substrate was further etched in inductively coupled HBr plasma. Finally, the hard mask was etched in buffered HF.

### Electrical measurements

Measurements were performed in a dilution refrigerator with a base temperature of 20 mK. Critical currents *I*_C_ were measured by applying a sawtooth wave *I*_SD_ signal with an amplitude of 49 μA and repetition rates between 33 and 133 Hz, whereas voltages *V* across the nanowires were recorded by a digital oscilloscope. The measurement setup was synchronized so that a switch from zero to a finite voltage in the oscilloscope could be related to the source–drain current at which the switch occurred. This technique allowed us to reliably extract critical currents down to 700 nA. The critical currents presented in Fig. 1 were obtained by averaging 108 such switching events. Sporadic fluctuations of *I*_C_ visible at *T* ≥ 1.5 K are associated with the instabilities of the temperature controller. The SPDs presented in Fig. 4 were obtained by recording 20,000 switches over a time interval of 10 min. To keep the nanowire potential constant as *I*_SD_ varies, *I*_SD_ was generated by sourcing two synchronized sawtooth waves with opposite polarities into 163 kΩ resistors placed at both ends of the nanowire (which add to the existing line resistance of 2.2 kΩ). Gate voltages were applied via high-precision source-measure units, which recorded the current flowing into the gate contacts. The gate current data shown in Fig. 1e,h were obtained after subtracting the linear components ranging between 1 and 5 pA V^−1^, as discussed elsewhere^9^. Such resistive contributions are attributed to spurious leakage paths in our setup.

### Electrostatic simulations

The electric-field distributions presented in Fig. 1 were produced with finite-element 3D electrostatic simulations performed with Ansys Maxwell version 2019R2. A substrate permittivity of 12 was assumed to resemble the electromagnetic properties of silicon, and its thickness was set to 1 μm. The metallic layer comprising the nanowire and gate electrodes was modelled as a 20-nm-thick perfect conductor. The upper edges of the structures were filleted with a radius of 3 nm. The geometry of the nanowire and gates was generated from the same layout file used for the electron-beam lithography of the devices. The fields shown in Fig. 1b,f are slices of the 3D simulation taken at half the height of the nanowire. Figure 1c shows the image taken perpendicular to the substrate and intersecting the gate electrode. The colour scale was saturated to a maximum value of 70 MV m^−1^ to evidence the field distribution, whereas the full scale reached up to 500 MV m^−1^.

## Supplementary information

Supplementary Information Supplementary Sections 1–5 and Figs. 1–5.

## Data Availability

The data presented in this work are available at 10.5281/zenodo.5825804. Further data that support the findings of this study are available from the corresponding authors upon reasonable request.
