# Supplementary material for: Out-of-equilibrium phonons in gated superconducting switches
Source: Nat Electron. 2022 Feb 28;5(2):71–7. doi: 10.1038/s41928-022-00721-1 (PMC8885403; doi:10.1038/s41928-022-00721-1)
Supplement: Supplementary file 1 — Supplementary Sections 1–5 and Figs. 1–5. [file 41928_2022_721_MOESM1_ESM.pdf]

nature portfolio

https://doi.org/10.1038/s41928-022-00721-1

Supplementary information

Out-of-equilibrium phonons in gated superconducting switches

In the format provided by the
authors and unedited

Supplementary Information: Out-of-equilibrium phonons in gated superconducting switches

M. F. Ritter,[1] N. Crescini,[1] D. Z. Haxell,[1] M. Hinderling,[1] H. Riel,[1] C. Bruder,[2] A. Fuhrer,[1,*] and F. Nichele[1,†]

[1] *IBM Quantum, IBM Research - Zurich, Säumerstrasse 4, 8803 Rüschlikon, Switzerland*

[2] *Department of Physics, University of Basel, Klingelbergstrasse 82, CH-4056 Basel, Switzerland*

(Dated: January 12, 2022)

SUPPORTING INFORMATION 1: DEVICE A2

Device A2 was lithographically equivalent to Device A1, shown in Fig. 1(a) of the Main Text, except for the distance d between gates 2,3 and the nanowire ($d = 1$ μm in Device A1 and $d = 80$ nm in Device A2). A false color image of Device A2 is shown in Fig. S.1, together with a simplified measurement schematic. The nanowire under study is depicted blue, the three gates red. Critical current I_C and gate current I_{G1} as a function of V_{G1} are shown in Fig. S.1(b) and (c) respectively. Their characteristics are almost identical to those of Device A1 (see Fig. 1(d,e) of the Main Text). The dependence on $V_{G2} - V_{G3}$ is depicted in Figs. S.1(d) and (e). Suppression of I_C was again correlated with the increase of the current I_{G2}. This time, sweeping gates 2 and 3 at the same voltage resulted in a partial suppression of I_C (gray line in Fig. S.1(d) shows I_C as a function of the parameter $V_S = 2V_{G2} = 2V_{G3}$) correlated with currents flowing from gates 2 and 3 into the nanowire (gray line in Fig. S.1(e)). The fact that I_C was affected at higher voltages for equal gate biases $V_{G2} = V_{G3}$ with respect to the asymmetric bias configuration ($V_{G2} = -V_{G3}$) (markers) speaks against any effect linked to electric fields between gates and nanowire.

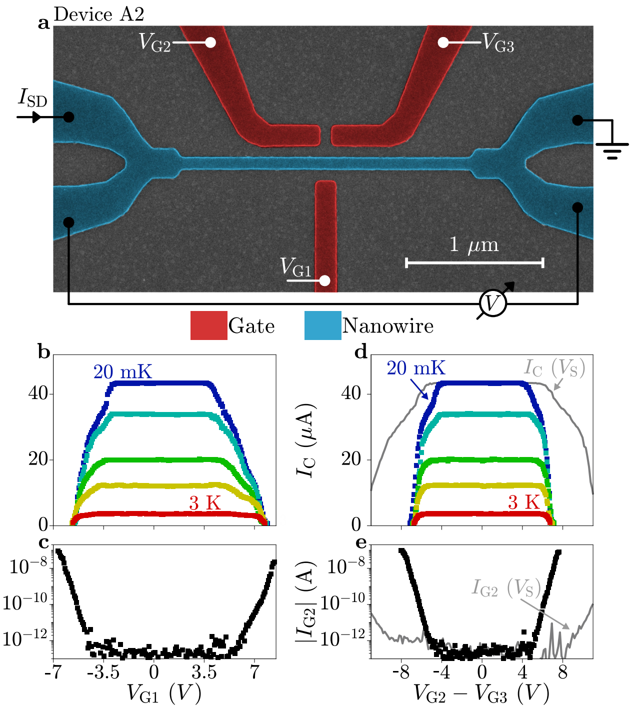

FIG. S.1. **Basic characterization of Device A2** (a) False-color scanning electron micrograph of Device A2 and simplified measurement configuration. The nanowire under investigation is depicted blue and the gates red. (b) Critical current I_C as a function of gate voltage V_{G1} at temperatures T of 20 mK (blue), 1.5 K, 2.1 K, 2.5 K and 3.0 K (red). (c) Gate current I_{G1} as a function of V_{G1} at $T = 20$ mK. (d) Critical current I_C as a function of gate voltage difference $V_{G2} - V_{G3}$ for the same temperature values as in (b) (markers) together with I_C as a function of the parameter $V_S = 2V_{G2} = 2V_{G3}$. (e) Gate current I_{G2} as a function of gate voltage difference $V_{G2} - V_{G3}$ measured at $T = 20$ mK (black markers), together with the current I_{G2} as a function of V_S.

SUPPORTING INFORMATION 2: DEVICES B AND C

In the Main Text we show a summary of the measurements obtained with Devices B and C using parametric plots of I_C as a function of I_{G1} and I_{G2}. Figure S.2 shows the datasets from which these parametric plots are obtained. Measurements were performed at temperatures ranging from 20 mK (blue) to 3 K (red). Gate currents are reported only for 20 mK.

SUPPORTING INFORMATION 3: REFERENCE DEVICES AFTER ADDITIONAL FABRICATION

In the Main Text we noted that devices which underwent additional fabrication steps, showed changes in some of their properties. Here we discuss this in more detail. In order to etch the trench into the Si substrate (see Fig. 2(a) of the Main Text) the entire sample was covered by a hard mask comprising a 2 nm thick Si_3N_4 layer, grown by plasma enhanced atomic layer deposition, and a 210 nm thick SiO_2 layer grown by plasma enhanced chemical vapor deposition. Both depositions were performed at a temperature of 300 °C. After definition of the trench by electron beam lithography, reactive ion etching and inductively coupled plasma etching, the hard mask was removed by immersion in buffered HF. While the trench was etched only in Device B, additional reference devices (RDs) on this chip underwent the same

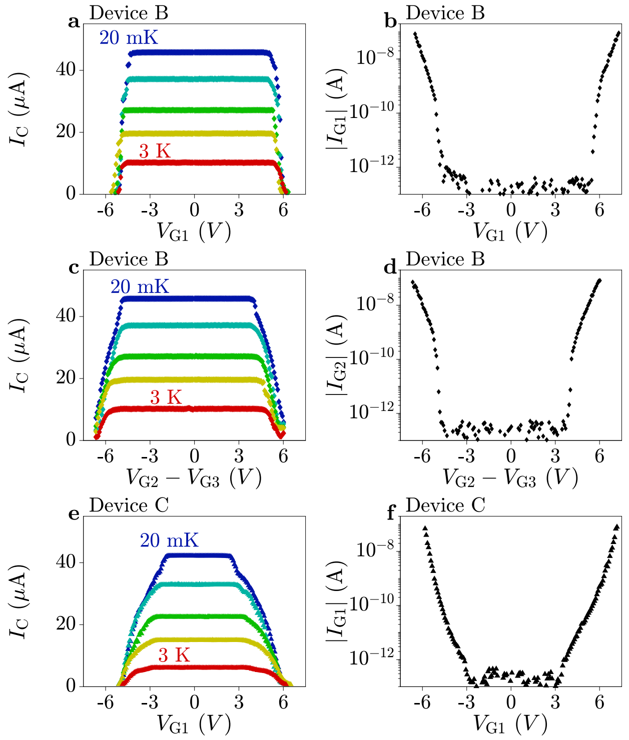

FIG. S.2. **Basic characterization of Devices B and C** (a) Critical current I_C of Device B as a function of gate voltage V_{G1} at temperatures T of 20 mK (blue), 1.5 K, 2.1 K, 2.5 K and 3.0 K (red). (b) Gate current I_{G1} of Device B as a function of V_{G1} at $T = 20$ mK. (c) Critical current I_C as a function of gate voltage difference $V_{G2} - V_{G3}$ of Device B for the same temperature values as in (a). (d) Gate current I_{G2} as a function of gate voltage difference $V_{G2} - V_{G3}$ measured at $T = 20$ mK. (e) Critical current I_C of Device C as a function of gate voltage V_{G1} for the same temperature values as in (a). (f) Gate current I_{G1} of Device C as a function of V_{G1} at $T = 20$ mK.

deposition and etching of the hard mask. We refer to these RDs as RD 1, RD 2 and RD 3, respectively. Reference devices had a similar geometry to Device A1, with $d = 1$ μm, 800 nm and 400 nm, respectively.

Figure S.3(a) shows a parametric plot of I_C as a function of I_{G1} for all devices that underwent deposition and etching of the Si_3N_4/SiO_2 hard mask, plus Device A1. The trench in the Si substrate was etched only for Device B. All devices that underwent further processing showed a characteristic asymmetric behavior, with I_C decreasing faster for $I_{G1} < 0$ than for $I_{G1} > 0$. We also found that RDs exhibited a reduced suppression efficiency for $I_{G1} > 0$. Figure S.3(b) shows a parametric plot of I_C as a function of I_{G2}. We notice that Device A1, which did not undergo additional fabrication, showed the fastest suppression of I_C. Reference Devices 1, 2 and 3 have quantitatively similar behavior, despite the fact that d varies from 400 nm to 1 μm. This is presumably due to natural sample-to-sample variations following the ad-

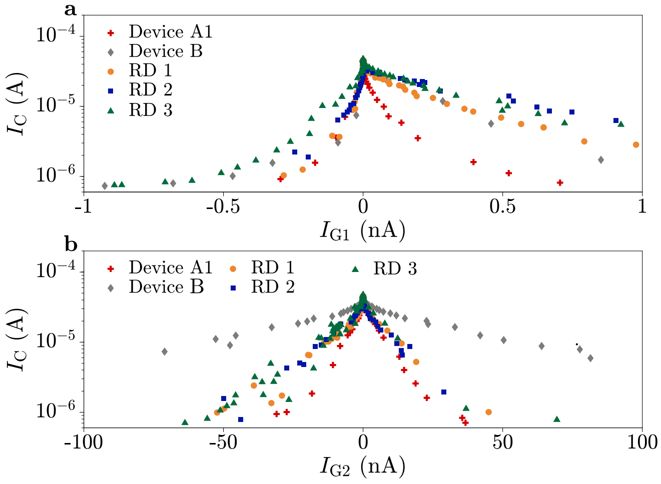

FIG. S.3. **Comparison to reference devices** (a) Critical current I_C as a function of gate current I_{G1} for Device A1, Device B, and three reference devices (RDs). All devices except Device A1 underwent additional fabrication. (b) Critical current I_C as a function of remote gate current I_{G2} for the same devices as in (a). Device B is the only one with a trench between gates and nanowire.

ditional fabrication. It makes extraction of a dependence on d difficult with just these three RDs. However, Device B clearly stands out from the rest, indicating that the presence of the etched trench in the substrate causes a significant suppression of the long distance coupling between current I_{G2} and nanowire. This clearly substantiates our explanation based on phonons.

SUPPORTING INFORMATION 4: COMPARISON OF POWER DEPENDENCES

Figure 3 of the Main Text compares the suppression of I_C in different devices as a function of injected power and for $T = 20$ mK. Similar curves are shown in Figure S.4 for Devices A1 and B at various temperatures, with panels (a) to (c) summarizing the behavior as a function of V_{G1}, and panels (d) to (f) summarizing the behavior as a function of $V_{G2} - V_{G3}$. The power $P_{2\mu A}$, needed to reach $I_C = 2$ μA via Gate 1, is plotted in Fig. S.4(c) as a function of temperature. As stated in the Main Text, the configuration with $V_{G1} < 0$ requires 2.5 times less power than the one with $V_{G1} > 0$ for low temperatures. We further show here that the asymmetry tends to vanish as the critical temperature is approached, with a temperature dependence reminiscent of the superconducting order parameter. Figure S.4(f) shows the temperature dependence of $P_{5\mu A}$, the power needed to reach $I_C = 5$ μA via Gate 2 and 3. The green (blue) curve is for Device A1 (Device B) respectively. For Device B with a trench between remote gates and nanowire $P_{5\mu A}$ is roughly six times larger than for Device A1, but both

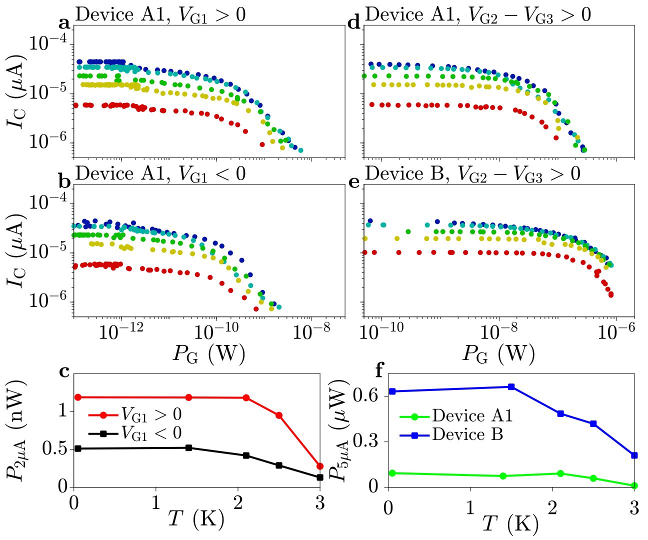

FIG. S.4. **Temperature dependence of the switching power** (a) I_C in Device A1 as a function of power $P_G = V_{G1} I_{G1}$, for $V_{G1} > 0$ for temperatures T of 20 mK (blue), 1.5 K, 2.1 K, 2.5 K and 3 K (red). (b) As in (a), but for $V_{G1} < 0$. (c) Power needed to reach $I_C = 2\ \mu$A with Gate 1 as a function of temperature. (d) I_C in Device A1 as a function of power $P_G = (V_{G2} - V_{G3}) I_{G2}$, for $(V_{G2} - V_{G3}) > 0$. Temperatures as in (a). (e) As in (d), but for Device B. (f) Power needed to reach $I_C = 5\ \mu$A with a gate voltage difference $V_{G2} - V_{G3}$ as a function of temperature.

curves decrease with temperature similar to the situation with local gate electrodes.

SUPPORTING INFORMATION 5: FIT OF THE SWITCHING PROBABILITY DISTRIBUTION

Figure 4 of the Main Text shows the switching probability distribution (SPD) measured in Devices A1 and C. Such measurements were performed by ramping the source drain current I_{SD} and recording, for each sweep, the I_{SD} value where switching from superconducting to resistive state occurred. Data was acquired for 2×10^4 switching events and I_{SD} was ramped at a rate $v = 6.4$ mAs^{-1}. In Fig. S.5(a) we plot again the SPDs measured in Device A1 at zero gate voltages and at a temperature of 20 mK (blue circles) and 2.2 K (green circles). In Fig. S.5(b) we plot the switching rates (markers), obtained from the data in Fig. S.5(a) by KFD transform [1]:

$$\Gamma(I_{SD}) = vP(I_{SD}) \left(1 - \int_0^{I_{SD}} P(I) dI\right)^{-1}, \qquad \text{(S.1)}$$

where P is the measured switching probability.

We fit to the SPD for each temperature, as shown in Fig. S.5(a), to a model for the switching rate of a superconducting nanowire [2] that follows the relation:

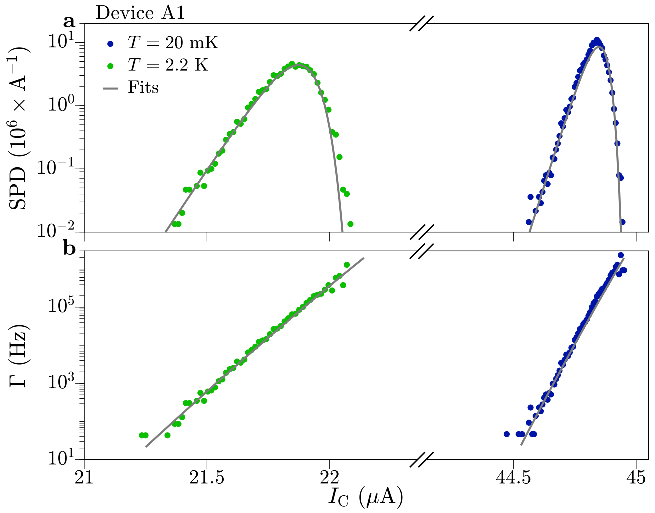

FIG. S.5. **Fitting of the switching probability distributions** (a) Switching probability distribution in Device A1, measured at temperatures T of 20 mK (blue circles) and 2.2 K (green circles) together with fits of a theory of phase escape via macroscopic quantum tunneling and thermal activation (solid gray lines). (b) Phase particle escape rates Γ of the same data as in (a), calculated with Eq. S.1 (markers), together with calculations of the escape rate using Eq. S.2, using the fit parameters obtained from (a).

$$\Gamma(I_{SD}, T) = \Omega(I_{SD}, T) \left[e^{-U(I_{SD},T)/T} + e^{-U(I_{SD},T)/T_q}\right], \qquad \text{(S.2)}$$

where the attempt frequency is $\Omega(I_{SD}, T) = \Omega_0 \left(1 - T^2/T_C^2\right)^{3/4} \left(1 - I_{SD}/I_C(T)\right)^{\nu}$ and the potential barrier height is $U(I_{SD}, T) = \frac{\kappa \hbar I_C(T)}{e} \left(1 - I_{SD}/I_C(T)\right)^{\eta}$. I_C is considered to follow Bardeen's formula: $I_C(T) = I_{C0} \left(1 - T^2/T_C^2\right)^{3/2}$. The temperature dependence of the attempt frequency follows from $\Omega(I_{SD}, T) \propto I_C^{1/2}$. For a nanowire forming a phase slip junction, we have $\kappa = \sqrt{6}/2$, $\eta = 5/4$ and $\nu = 5/8$. This model accounts for switching due to macroscopic quantum tunneling (MQT) and thermally activated phase escape mechanisms, which dominate at low and high temperatures respectively. We convert the modeled switching rate Γ to a SPD using the inverse KFP transform [1]:

$$P(I_{SD}) = \frac{\Gamma}{v} \exp\left(-1/v \int_0^{I_{SD}} \Gamma(I) dI\right). \qquad \text{(S.3)}$$

We fit the logarithm of the probability distribution to optimally account for the shape of the SPD tails. We set $T_C = 3.7$ K, as measured in Ref. [3], and fit with Ω_0, I_{c0} and T_q as free parameters. From the fit, we obtain $\Omega_0 = 67.5 \times 10^{12}$ rads^{-1}, $I_{c0} = 45.7\ \mu$A, $T_q = 0.77$ K. The finding of $T_q \gg 20$ mK confirms MQT

is the dominant phase escape mechanism at low temperatures. With these parameters, one should be able to calculate the SPD at any given temperature. However, we find that the curve at $T = 2.2$ K is satisfactorily reproduced only by setting the parameter κ to 71% of its theoretical value, similar to previous observations [2]. The value $\kappa = \sqrt{6}/2$ was derived under the assumption of a nanowire width much larger than the superconducting coherence length [4]. This assumption might not be completely justified in the current experiment.

Alternatively to the fit, it is possible to relate the standard deviation of the switching currents σ_{I_C} to an effective energy $E_\mathrm{eff} = k_B T_\mathrm{eff}$ via the Kurkijavi power law [5]:

$$\sigma_{I_\mathrm{C}} \propto I_\mathrm{C}^{1/3} \left(\frac{k_B T_\mathrm{eff}}{\Phi_0} \right)^{2/3} \tag{S.4}$$

where $\Phi_0 = h/2e$ is the flux quantum. In the Main Text we use such a relation to extract an effective temperature from the broad SPDs resulting from current injection in the nanowires.

* afu@zurich.ibm.com
† fni@zurich.ibm.com

[1] A. Bezryadin, Stochastic premature switching and Kurkijärvi theory, in *Superconductivity in Nanowires* (John Wiley & Sons, Ltd, 2012) Chap. 8, pp. 131–162.

[2] A. Murphy, P. Weinberg, T. Aref, U. C. Coskun, V. Vakaryuk, A. Levchenko, and A. Bezryadin, Phys. Rev. Lett. **110**, 247001 (2013).

[3] M. F. Ritter, A. Fuhrer, D. Z. Haxell, S. Hart, P. Gumann, H. Riel, and F. Nichele, Nat. Commun. **12**, 1266 (2021).

[4] M. Tinkham and C. N. Lau, Appl. Phys. Lett. **80**, 2946 (2002).

[5] A. Bezryadin, *Superconductivity in Nanowires: Fabrication and Quantum Transport* (Wiley-VCH Verlag GmbH & Co. KGaA, 2012).
